# Influence of hydration status on cardiovascular magnetic resonance myocardial T1 and T2 relaxation time assessment: an intraindividual study in healthy subjects

**DOI:** 10.1186/s12968-020-00661-9

**Published:** 2020-09-07

**Authors:** Julian A. Luetkens, Marilia Voigt, Anton Faron, Alexander Isaak, Narine Mesropyan, Darius Dabir, Alois M. Sprinkart, Claus C. Pieper, Johannes Chang, Ulrike Attenberger, Daniel Kuetting, Daniel Thomas

**Affiliations:** 1 Department of Diagnostic and Interventional Radiology, University Hospital Bonn, Venusberg-Campus 1, 53127 Bonn, Germany; 2 Quantitative Imaging Lab Bonn (QILaB), Bonn, Germany; 3 Department of Internal Medicine I, University Hospital Bonn, Venusberg-Campus 1, 53127 Bonn, Germany

**Keywords:** T1 mapping, T2 mapping, Dehydration, Fluid status, Confounder

## Abstract

**Background:**

Myocardial native T1 and T2 relaxation time mapping are sensitive to pathological increase of myocardial water content (e.g. myocardial edema). However, the influence of physiological hydration changes as a possible confounder of relaxation time assessment has not been studied. The purpose of this study was to evaluate, whether changes in myocardial water content due to dehydration and hydration might alter myocardial relaxation times in healthy subjects.

**Methods:**

A total of 36 cardiovascular magnetic resonance (CMR) scans were performed in 12 healthy subjects (5 men, 25.8 ± 3.2 years). Subjects underwent three successive CMR scans: (1) baseline scan, (2) dehydration scan after 12 h of fasting (no food or water), (3) hydration scan after hydration. CMR scans were performed for the assessment of myocardial native T1 and T2 relaxation times and cardiac function. For multiple comparisons, repeated measures ANOVA or the Friedman test was used.

**Results:**

There was no change in systolic blood pressure or left ventricular ejection fraction between CMR scans (*P* > 0.05, respectively). T1 relaxation times were significantly reduced with dehydration (987 ± 27 ms [*baseline*] vs. 968 ± 29 ms [*dehydration*] vs. 986 ± 28 ms [*hydration*]; *P* = 0.006). Similar results were observed for T2 relaxation times (52.9 ± 1.8 ms [*baseline*] vs. 51.5 ± 2.0 ms [*dehydration*] vs. 52.2 ± 1.9 ms [*hydration*]; *P* = 0.020).

**Conclusions:**

Dehydration may lead to significant alterations in relaxation times and thereby may influence precise, repeatable and comparable assessment of native T1 and T2 relaxation times. Hydration status should be recognized as new potential confounder of native T1 and T2 relaxation time assessment in clinical routine.

## Background

Myocardial native T1 and T2 mapping allow for a non-invasive quantification of myocardial tissue alterations across a broad range of myocardial diseases in patients eligible for cardiovascular magnetic resonance (CMR) imaging [1]. As myocardial mapping is very sensitive for the detection of myocardial tissue abnormalities like myocardial edema or fibrosis, current guidelines (e.g. for inflammatory cardiomyopathies) already recommend the implementation of mapping techniques into clinical routine [2]. However, it is important to know that mapping techniques can be prone to confounders such as heart rate and magnetic field inhomogeneities [1, 3, 4]. Apart from these variations caused by confounders, there are also subtle differences in myocardial T1 and T2 relaxation times that are related to gender or age [5]. Moreover, myocardial iron content can drastically alter myocardial relaxation times [4]. Although it is well known that myocardial T1 and T2 relaxation are sensitive to pathological increase of myocardial water content (e.g. myocardial edema) [6–8], the influence of physiological hydration changes as a possible confounder of relaxation time assessment has not been fully evaluated yet. Although previous experimental studies suggest that changes in CMR relaxation times occur with non-pathological alterations in myocardial water content [9], no actual in vivo data is available about the relationship between changes in myocardial water content and concordant myocardial native T1 and T2 relaxation time measurements. Thus, the purpose of this study was to evaluate whether the change in myocardial water content due to dehydration and hydration might alter the correct assessment of myocardial relaxation times in healthy subjects.

## Materials and methods

**Fig. 1:**
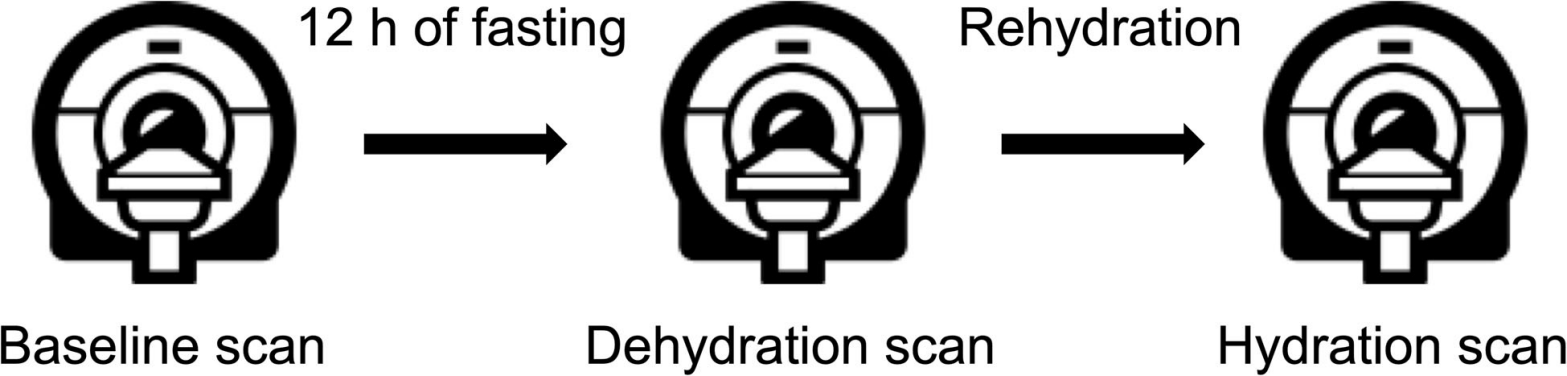
Study protocol. Healthy subjects were included in this study to undergo three separate cardiovascular magnetic resonance (CMR) studies. For each participant, all CMR scans were performed on the same day. After the baseline scan in the morning hours, the subjects fasted for 12 h (no food and no water). Within these 12 h the subjects had their normal daily routine. In the evening, a dehydration scan was performed. Afterwards, the participant had access to a sandwich and water, and they were advised to drink at least 1.5 l of water. After drinking this amount, the final hydration scan was performed. On every CMR scan, native T1 and T2 mapping was acquired

The institutional review board approved this prospective study and all subjects gave written informed consent. The study population consisted of healthy subjects without known cardiovascular disease and without cardiovascular disease risk factors. All subjects had an unremarkable past medical history of cardiovascular disease. Electrocardiographic results prior to CMR were unremarkable. All control participants had normal CMR results without structural abnormalities or wall motion abnormalities. To assess possible influences of hydration status on native T1 and T2 relaxation time measurements, three separate CMR scans were performed in each participant: The baseline scan (*normal scan*) was performed during the morning hours. Participants were allowed to have normal breakfast at home, but coffee intake was restricted. Also, they were told not to ingest significant amounts of water prior to the baseline scan. After 12 h of fasting (no access to food or water) another scan was performed (*dehydration scan*). The last scan was performed after adequate hydration (*hydration scan*). After the end of fasting each participant was offered a standardized small cheese sandwich. For hydration, the participants were instructed to drink 1.5 l of still water within 15 min. Drinking was supervised by a study team member. The hydration scan was started 15 min after fluid intake to allow water absorption. For all participants the scans were performed at 1 day. Blood pressure measurements and laboratory markers were obtained directly prior to every scan. Blood samples were promptly transported to the central laboratory and were immediately analyzed. A visualized description of the study protocol is presented in Fig. 1. For the assessment of inter-study reproducibility another group of healthy subjects was studied. Inter-study reproducibility was measured from two separate examinations that were acquired within two consecutive CMR examinations during the same day.

### CMR

All imaging was performed on a clinical whole-body 1.5 T CMR system (Ingenia; Philips Healthcare, Best, the Netherlands). A 32-channel torso coil with digital interface was used for signal reception. For functional and myocardial strain analysis, electrocardiographic-gated balanced steady-state free precession cine images were obtained in short-axis, four-chamber, and two-chamber views. Scan parameters for short-axis cine imagines were: time of repetition 2.8 ms, time of echo 1.38 ms, flip angle 60°, parallel imaging factor 3, acquired voxel size 1.79 × 2 × 8 mm, reconstructed voxel size 0.99 × 0.99 × 8 mm, scan duration/breath hold 13.0 s, cardiac phases per RR interval 40. Native T1 and T2 mapping were performed in end-diastole in short-axis views. For myocardial T1 and T2 mapping, one midventricular section was acquired. For myocardial T1 mapping, an optimized native 5(3 s)3 modified Look-Locker inversion recovery (MOLLI) acquisition scheme was used [10]. Following parameters were applied: time of repetition 2.1 ms, time of echo 0.96 ms, flip angle 35°, parallel imaging factor 2, acquired voxel size 1.97 × 2.46 × 10 mm, reconstructed voxel size 1.17 × 1.17 × 10 mm, scan duration/breath hold 11.7 s. For myocardial T2 mapping, a six-echo gradient spin-echo sequence was used [11] with application of the following parameters: time of repetition 1 RR interval, time of echo 23.6/∆TE = 11.8 (6Ec), flip angle 90°, parallel imaging factor 2, acquired voxel size 1.97 × 2.03 × 10 mm, reconstructed voxel size 1.03 × 1.03 × 10 mm, scan duration/breath hold 14.0 s. In cases of motion artifacts on T1 and T2 mapping images, sequences were repeated until a sufficient image quality was achieved. No contrast was administered.

### Image analysis

Image analysis was performed by two radiologists experienced in CMR (J.A.L. (reader 1) and D.T. (reader 2)). Readers were blinded to the scan and personal data. CMR analyses were performed offline by using dedicated software (IntelliSpace Portal, version 10.1; Philips Healthcare). Myocardial native T1 and T2 relaxation maps were reconstructed offline by using a software-implemented motion-correction algorithm (fast elastic image registration). Myocardial contours were drawn throughout the T1 and T2 maps to investigate the entire midventricular myocardium, and mean midventricular T1 and T2 relaxation times were calculated. For intra- and inter-reader reproducibility measurements all relaxation time measurements were performed by reader 1 and 2 and were repeated by reader 1. Inter-study reproducibility measurements were performed by reader 1.

### Statistical analysis

Prism (version 7.0d; GraphPad Software, La Jolla, California, USA) and SPSS Statistics (version 23; Statistical Package for the Social Sciences, International Business Machines, Inc., Armonk, New York, USA) were used for statistical analysis. Subject characteristics are presented as mean ± standard deviation or as absolute frequency. Kolmogorov-Smirnov test was used for the assessment of normality. Continuous variables between two groups were compared by using Student *t* test. For multiple comparisons, repeated measures one-way analysis of variance (ANOVA) followed by Tukey’s multiple comparison test or the nonparametric equivalent (Friedman test followed by Dunn’s multiple comparisons test) were used where appropriate. Intra- and inter-rater reproducibility of T1 and T2 mapping measurements was assessed using intraclass correlation coefficient (ICC) estimates. ICC estimates and their 95% confident intervals (CI) were based on a single measurement, absolute-agreement, 2-way mixed-effects model. Single measure coefficients are reported. Bland Altman analysis was also used to assess agreement and bias among T1 and T2 mapping measurements. The level of statistical significance was set to *P* < 0.05.

## Results

A total of 12 healthy subjects were enrolled (5 male). Mean age was 25.8 ± 3.2 years (men: 28.0 ± 3.2; range 25–32; women: 24.3 ± 2.1; range 24–26; *P* = 0.063). Mean body mass index was 22.9 ± 3.5 kg/m^2^ (mean height: 175.2 ± 8.9 cm, mean weight: 70.6 ± 12.8 kg). All participants completed the study protocol. Structural CMR results were normal for all participants.

### Laboratory results

As a sign of the lack of water intake, we found significantly increased levels of white blood cell count (5.8 ± 0.7 G/l [*baseline*] vs. 7.4 ± 2.0 G/l [*dehydration*] vs. 6.9 ± 1.4 G/l [*hydration*]; *P* = 0.003), red blood cell count (5.0 ± 0.4 G/l [*baseline*] vs. 5.1 ± 0.5 G/l [*dehydration*] vs. 5.0 ± 0.5 G/l [*hydration*]; *P* = 0.021), and platelet count (238 ± 43 G/l [*baseline*] vs. 255 ± 56 G/l [*dehydration*] vs. 241 ± 50 G/l [*hydration*]; *P* = 0.009) in dehydration. Hematocrit levels were significantly elevated during the dehydration scan (41.8 ± 3.0% [*baseline*] vs. 43.0 ± 3.6% [*dehydration*] vs. 41.7 ± 3.8% [*hydration*]; *P* = 0.021). Also, magnesium electrolyte level was increased in dehydration (0.8 ± 0.1 mmol/l [*baseline*] vs. 0.9 ± 0.0 mmol/l [*dehydration*] vs. 0.8 ± 0.0 mmol/l [*hydration*]; *P* = 0.002). All other laboratory results are given in the Table 1.

**Table 1:** Clinical, laboratory and cardiovascular magnetic resonance (CMR) parameters for the baseline, dehydration and hydration scan

Variable	Baseline scan (***n*** = 12)	Dehydration scan (***n*** = 12)	Hydration scan (***n*** = 12)	***P*** value
*Clinical parameters*				
Systolic blood pressure (mmHg)	124 ± 12	122 ± 10	122 ± 9	0.520
Diastolic blood pressure (mmHg)	69 ± 7	72 ± 9	67 ± 7	0.212
Heart rate (bpm)	63 ± 6^§^	59 ± 11^*^	64 ± 10	**0.014**
*Laboratory parameters*				
Sodium (mmol/l)	140 ± 2	141 ± 3	140 ± 3	0.210
Potassium(mmol/l)	4.3 ± 0.5	4.1 ± 0.4	4.0 ± 0.5	0.125
Calcium (mmol/l)	2.4 ± 0.1	2.4 ± 0.1	2.4 ± 0.1	0.175
Chloride (mmol/l)	102 ± 2	102 ± 3	101 ± 3	0.147
Magnesium (mmol/l)	0.8 ± 0.1^§^	0.9 ± 0.1^*‡^	0.8 ± 0.1^§^	**0.002**
Creatinine (mg/dl)	0.8 ± 0.1	0.8 ± 0.1	0.8 ± 0.1	0.226
White blood cell count (G/l)	5.8 ± 0.7^§^	7.4 ± 2.0^*^	6.9 ± 1.4	**0.003**
Red blood cell count (G/l)	5.0 ± 0.4	5.1 ± 0.5^‡^	5.0 ± 0.5^§^	**0.021**
Hemoglobin (g/dl)	14.5 ± 1.1	14.7 ± 1.1	14.5 ± 1.2	0.184
Hematocrit (%)	41.8 ± 3.0	43.0 ± 3.6^‡^	41.7 ± 3.8^§^	**0.021**
Platelet count (G/l)	238 ± 43^§^	255 ± 56^*‡^	241 ± 50^§^	**0.009**
*Cardiac MRI parameters*				
Left ventricular ejection fraction (%)	64.8 ± 3.8	62.3 ± 3.7	66.9 ± 4.5	0.179
Left ventricular end-diastolic volume (ml)	141.3 ± 28.7	142.9 ± 35.7	149.2 ± 35.7	0.076
Left ventricular end-diastolic volume index (ml/m^2^)	78.5 ± 13.1	76.2 ± 12.8^§^	80.3 ± 12.9^‡^	**0.009**
End-diastolic wall mass (g)	82.3 ± 20.2	80.2 ± 24.3	88.91 ± 26.2	**0.010**
Right ventricular ejection fraction (%)	63.0 ± 6.4	59.5 ± 5.9	62.4 ± 7.6	0.420
Right ventricular end-diastolic volume (ml)	151.2 ± 36.7	144.9 ± 36.4	148.8 ± 38.0	0.558
Right ventricular end-diastolic volume index (ml/m^2^)	81.5 ± 14.5	78.0 ± 14.2	80.2 ± 15.3	0.558
Native T1 relaxation time (ms)	987 ± 27^‡^	968 ± 29^*§^	986 ± 28^‡^	**0.006**
T2 relaxation time (ms)	52.9 ± 1.8	51.5 ± 2.1	52.2 ± 1.9	**0.020**

Continuous variables are given as mean ± standard deviation

* *P* < .05 versus baseline scan

‡ *P* < .05 versus dehydration scan

§ *P* < .05 versus hydration scan

### CMR results

**Fig. 2:**
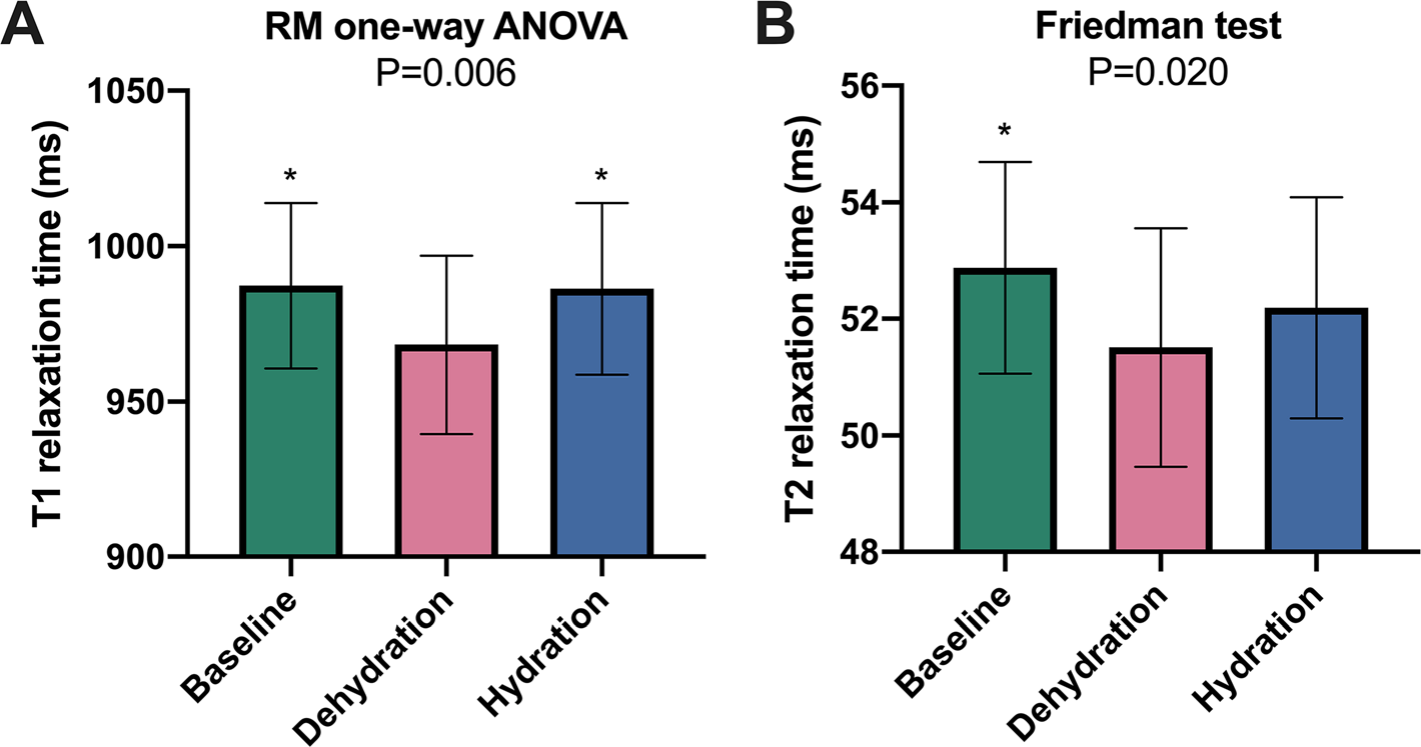
Column graphs show differences for T1 (**a**) and T2 relaxation times (**b**) in healthy subjects at baseline, after 12 h of fasting (“dehydration”) and after rehydration (“hydration”). Data are presented as mean with one standard deviation error bars. * indicates *P* < .05 against dehydration scan. RM = repeated measure

**Fig. 3:**
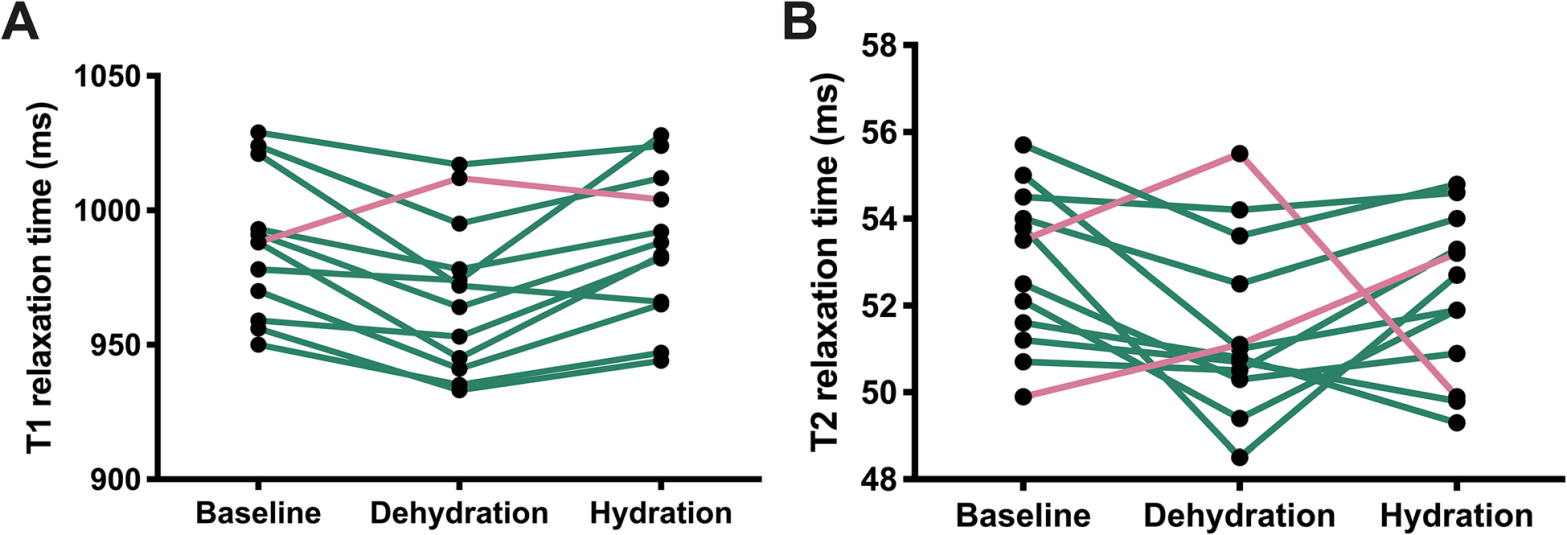
Individual plotted mean T1 (**a**) and T2 relaxation times (**b**) in healthy subjects at baseline, after 12 h of fasting (“dehydration”) and after rehydration (“hydration”). Connection lines of subjects with decreased values under dehydration are displayed in green. Connection lines of subjects with increased values under dehydration are displayed in pink

During the dehydration scan left ventricular end-diastolic volume index was significantly decreased (78.5 ± 13.1 ml/m^2^ [*baseline*] vs. 76.2 ± 12.8 ml/m^2^ [*dehydration*] vs. 80.3 ± 12.9 ml/m^2^ [*hydration*]; *P* = 0.009). Native T1 relaxation times were significantly reduced in dehydration (987 ± 27 ms [*baseline*] vs. 968 ± 29 ms [*dehydration*] vs. 986 ± 28 ms [*hydration*]; *P* = 0.006). Similar results were observed for T2 relaxation times (52.9 ± 1.8 ms [*baseline*] vs. 51.5 ± 2.0 ms [*dehydration*] vs. 52.2 ± 1.9 ms [*hydration*]; *P* = 0.020) (see Figs. 2 and 3).

**Fig. 4:**
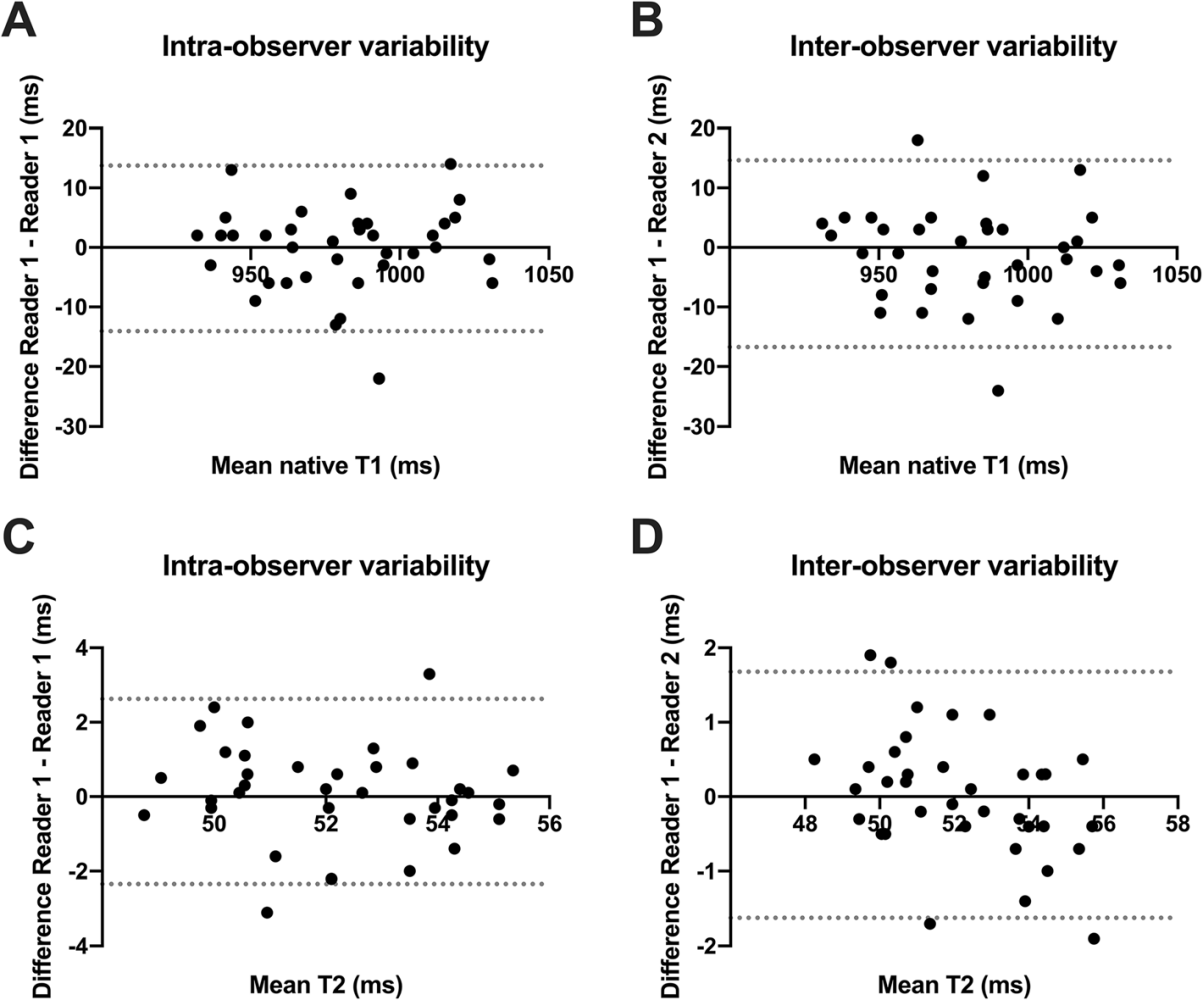
Bland-Altman plots for: intra-observer variability of mid-ventricular native T1 relaxation times (**a**), inter-observer variability of mid-ventricular native T1 relaxation times (**b**), intra-observer variability of mid-ventricular T2 relaxation times (**c**), and inter-observer variability of mid-ventricular T2 relaxation times (**d**)

**Fig. 5:**
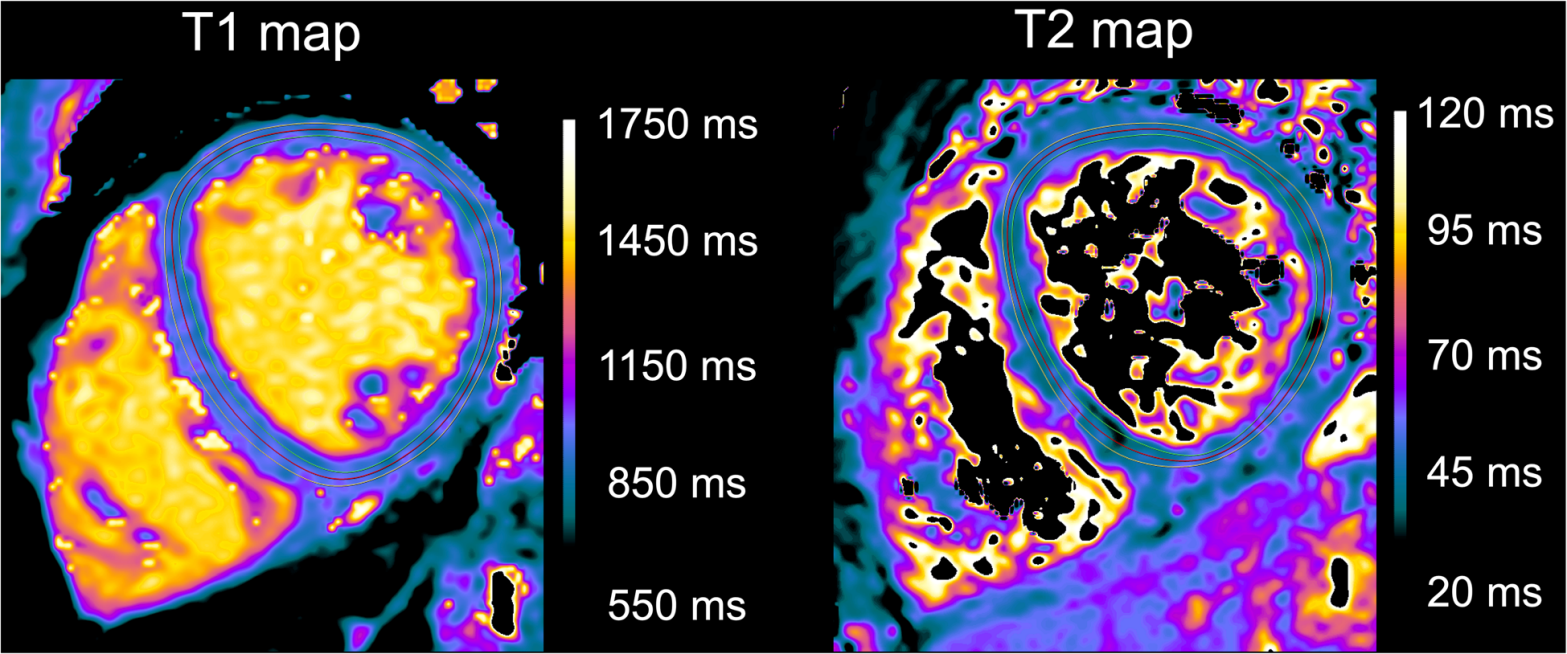
Exemplary region-of-interest (ROI) placement in a 26-year-old healthy subject after fasting. Midventricular section T1 and T2 map are provided. Subendocardial (green) and subepicardial (yellow) contours were drawn carefully throughout the entire midventricular myocardium. ROI placement was performed within the myocardium to avoid confounding partial volume effects of the blood pool and chemical shift artifacts. Mean T1 relaxation time was 933 ms and mean T2 relaxation time was 48.5 ms

Analysis of intra- and inter-rater reproducibility (ICC, single measures) of native T1 and T2 mapping measurements revealed good or excellent results: T1 (intra): 0.969, 95% CI: 0.940–0.984; T2 (intra): 0.808, 95% CI: 0.657–0.897; T1 (inter): 0.962 95%, CI: 0.926 to 0.980; T2 (inter): 0.924 95%, CI: 0.856 to 0.961. Bland Altman analysis showed a low bias for T1 (intra-observer: − 0.2 ± 7.1 ms (95% limits of agreement: − 14.1 - 13.7 ms); inter-observer: − 1.1 ± 8.0 ms (95% limits of agreement: − 16.7 - 14.6 ms)) and T2 measurements (intra-observer: 0.1 ± 1.2 ms (95% limits of agreement: − 2.3 - 2.6 ms); inter-observer: 0.0 ± 0.84 ms (95% limits of agreement: − 1.6 - 1.7 ms)) (see Fig. 4). Exemplary region of interest (ROI) placement is given in Fig. 5.

Inter-study reproducibility was assessed in another six healthy subjects, which were not part of the intervention group (4 men, 29.2 ± 2.5 years). Bland Altman analysis showed low bias for inter-study reproducibility (T1: 1.0 ± 6.6 ms (95% limits of agreement: − 11.9 - 13.8 ms); T2: − 0.6 ± 0.5 ms (95% limits of agreement: − 1.6 – 0.4 ms)).

## Discussion

In this prospective study, we evaluated whether differences in hydration status in healthy subjects could influence the assessment of myocardial native T1 and T2 relaxation times. The main finding of this study is that myocardial native T1 and T2 values were significantly reduced in dehydration, indicating an influence of hydration status on relaxation time assessment. Therefore, hydration status might be a confounder of native T1 and T2 mapping.

Dehydration (deficit of body water) is a general physiological condition that can have profound effects on human health [12]. Mild dehydration often only leads to slight impairment in physical or mental performance and can be easily corrected [13]. In our study we observed significantly increased levels of hematocrit and red as well as white blood cell count indicating measurable effect of dehydration. Also, left ventricular end-diastolic volume index was reduced due to the restrictions in fluid intake. Chronic dehydration can have serious effects on human health and might be associated with or might promote common public health disorders [14]. Especially, older adults are susceptible to dehydration due to age-related pathophysiological changes. When hospitalized, dehydrated older patients (aged ≥65 years) are 6 times more likely to die in hospital than patients with a normal level of hydration. However, in a clinical setting dehydration is often underdiagnosed. Uncomplicated dehydration (e.g. due to water deprivation) results in a significant loss of tissue water compared to normal hydrated controls, especially in the muscle and the skin [15].

Although different tissues with similar water content have been shown to have significantly different native T1 and T2 relaxation time [16], to some extent T1 and T2 relaxation time are a function of absolute tissue water content and correlate strongly with tissue water content [9, 17]. Investigating the same tissue (e.g. intervertebral disc anulus fibrosus and nucleus pulposus), correlations of relaxation times are mostly influenced by differences in tissue water content [17]. In a tissue analysis animal study from 1984, Brown et al. investigated T1 and T2 relaxation times of normal, volume overloaded, and dehydrated rabbit myocardium and found that native T1 and T2 values significantly correlated with percent tissue water content [9]. Compared to normal rabbits they found significantly reduced native T1 and T2 relaxation time in rabbit heart tissues, which were dehydrated by water restriction and furosemide-induced diuresis [9]. Our results indicate that also in healthy humans, differences in native T1 and T2 relaxation time can be measured depending on hydration status. Particularly in a dehydrated state, significant alterations in relaxation times compared to baseline measurements were observed. During dehydration myocardial relaxations times were reduced compared to baseline. Water loss due to water deprivation or inadequate water intake typically leads to hypertonic dehydration, which can be also caused by sweat loss (e.g. during exercise and fever) and osmotic diarrhea [12]. In hypertonic dehydration, water loss exceeds sodium loss, which is characterized by an osmotic shift of water from the intracellular to the extracellular compartment. The relatively lower amount of water in the intracellular compartment might have influenced myocardial native T1 and T2 relaxation time measurements observed in our study.

Although differences in myocardial native T1 relaxation times after dehydration compared to baseline were rather small (mean difference: 19 ± 19 ms), the mean difference was higher than the 95% of agreement of inter- and intra-observer and the inter-study reproducibility measurements, indicating a potential important effect when evaluating relaxation times in a clinical setting. However, the influence of dehydration in a clinical context has not been evaluated yet. According to a consensus statement about the clinical use of CMR mapping, local reference ranges for myocardial mapping should be obtained from healthy subjects and only values below or above two standard deviations should be considered as pathological [1]. The standard deviations for T1 mapping in this study ranged from 27 ms to 29 ms. In this regard, dehydration might not lead to pathological results in a predominant number of patients in a sample, but may possibly influence the result of the relaxation measurement in individual cases. Interestingly, during the hydration CMR we were not able to show increased myocardial relaxation times compared to baseline, possibly because the chosen intervention (drinking 1.5 l of water) was not sufficient to show a hyperhydration effect in the healthy subjects. In another CMR study using T1 mapping, Graham-Brown et al. investigated interstudy reproducibility in 10 hemodialysis patients and found T1 relaxation times to be unaffected from fluid status [18]. However, the study results cannot be generalized as no systematic scans (e.g. before and after dialysis) were performed in this study and the reported differences in Δ weight (as surrogate for the altered fluid status) were close to zero for the most patients. In our study, we showed decreased relaxation times healthy controls using strict study protocol and therefore random effects on fluid status, as in the study from Graham-Brown et al. [18], can be excluded. On the other hand, native T1 and T2 times are prolonged with increasing water content of tissue and the presence of myocardial edema, especially in myocardial infarction, myocarditis or other acute cardiomyopathies [6, 7, 19–21]. In this regard it is important to know that alterations in fluid status may in theory affect the assessment of relaxation times leading to confounding results. Hydration status might be an additional confounder the clinician should have in mind when interpreting results, especially in follow-up studies and in cases of subtle myocardial disease or fibrosis [22–25].

### Limitations

Because of the explorative study design, our study has several limitations. Our study population consisted of healthy young subjects with a low to normal body mass index. Therefore, our results may not be directly transferable to patient cohorts. Although the applied 5(3 s)3 MOLLI sequence has a reduced sensitivity to heart rate compared to the original MOLLI scheme [3], mean heart rate was decreased during dehydration. Although blood samples were standardized taken in the same sitting position prior to every CMR, recent studies suggest that hematocrit samples should be drawn at rest in supine position to avoid confounding by body posture [26]. Between the baseline scan and the dehydration scan, the subjects were not under observation and were not restricted in their daily routine. Therefore, differences in their daily behavior (e.g. physical exercise) could have had different strong influences on the extent of relaxation time alterations. In this regard, activities like endurance sport could have led to subtle myocardial edema [27], which might explain an increase of native T1 and T2 relaxation time observed in some participants during dehydration. Because of the limited sample size, the statistical evaluation of the results was only descriptive, and no additional regression analysis was performed. However, the reported pattern of decreased relaxation times and alterations of laboratory parameters during dehydration over several different variables underline the validity of the findings described.

## Conclusions

Hydration status may influence myocardial native T1 and T2 relaxation time. In our study, dehydration led to significant alterations in relaxation times and therefore may influence precise or repeatable assessment of native T1 and T2 relaxation times. Hydration status should be recognized as new potential confounder of T1 and T2 relaxation time assessment in clinical routine. Future studies should assess the influence of dehydration on CMR relaxation time measurements in a clinical setting.

## Data Availability

The datasets used and/or analyzed during the current study are available from the corresponding author on reasonable request.

## Abbreviations

CI: Confident interval
CMR: Cardiovascular magnetic resonance
ICC: Intraclass correlation coefficient
MOLLI: Modified Look-Locker inversion recovery
